# First Detection and Molecular Identification of *Rhabditis (Rhabditella) axei* from the Chinese Red Panda (*Ailurus styani*)

**DOI:** 10.3390/pathogens14080783

**Published:** 2025-08-06

**Authors:** Chanjuan Yue, Wanjing Yang, Dunwu Qi, Mei Yang, James Edward Ayala, Yanshan Zhou, Chao Chen, Xiaoyan Su, Rong Hou, Songrui Liu

**Affiliations:** The Conservation of Endangered Wildlife Key Laboratory of Sichuan Province, Chengdu Research Base of Giant Panda Breeding, 1375 Panda Road, Chenghua District, Chengdu 610081, China; chanjuan_yue@163.com (C.Y.); m15882669800@163.com (W.Y.); qidunwu@163.com (D.Q.); yangmei20230606@163.com (M.Y.); ayalajames@msn.com (J.E.A.); zhouyanshan_gsau@163.com (Y.Z.); ch7119@126.com (C.C.); suxiaoyan123456@sina.com (X.S.); hourong2000@panda.org.cn (R.H.)

**Keywords:** red panda, rhabditidae, nematode, morphological features, phylogenetic analyses

## Abstract

*Rhabditis* (*Rhabditella*) *axei* is a predominantly free-living nematode commonly found in sewage systems and decomposing organic matter. While primarily saprophytic, it has been documented as an opportunistic pathogen in human urinary and gastrointestinal tracts. The Chinese red panda (*Ailurus styani*), a rare and protected species in China, has not previously been reported as a host for *Rhabditis (Rhabditella)* spp. infections. This study reports the first documented occurrence of R. axei in red panda feces, unambiguously confirmed through integrative taxonomic approaches combining morphological and molecular analyses. The nematodes exhibited key morphological features consistent with *R. axei*, including a cylindrical rhabditiform esophagus, sexually dimorphic tail structures, and diagnostic spicule morphology. Molecular analysis based on 18S-ITS-28S rDNA sequencing confirmed their identity, showing >99% sequence similarity to *R. axei* reference strains (GenBank: PP135624.1, PP135622.1). Phylogenetic reconstruction using 18S rDNA and ITS rDNA sequences placed the isolate within a well-supported *R. axei* clade, clearly distinguishing it from related species such as *R. blumi* and *R. brassicae*. The findings demonstrate the ecological plasticity of *R. axei* as a facultative parasite capable of infecting non-traditional hosts and further highlight potential zoonotic risks associated with environmental exposure in captive wildlife populations. Our results emphasize the indispensable role of molecular diagnostics in accurately distinguishing morphologically similar nematodes within the Rhabditidae family, while providing essential baseline data for health monitoring in both in situ and ex situ conservation programs for this endangered species.

## 1. Introduction

*Rhabditis* (*Rhabditella*) *axei* (*R. axei*) is a free-living nematode commonly distributed in decomposing organic matter and sewage systems, occasionally exhibiting facultative parasitism in mammals. Under Dougherty’s (1955) classification system [1], *R. axei* is classified within the genus *Rhabditella*, which is regarded as a synonym of *Rhabditis*. After hatching from eggs, nematode larvae develop through molting stages to adulthood. Among the larval stages, the filariform stage is infectious. While *R. axei* is principally free-living, clinical cases confirm its capacity to complete life cycles within human and animal hosts [1]. Its facultative parasitic behavior, documented in humans, dogs, and poultry, reflects adaptive strategies that exploit compromised host defenses [1,2]. Although *R. axei* infections have been associated with clinical manifestations including hematuria and proteinuria in humans [3,4,5], its pathogenicity in novel hosts and its transmission dynamics remain poorly characterized, particularly in arboreal species such as the red panda.

The red panda, is a rare species of mammal endemic to the eastern Himalayas and the mountains of Southwest China. Recent research proposes that the red panda should be separated into two species, the Himalayan (*Ailurus fulgens*) and Chinese (*Ailurus styani*) red pandas, respectively [6]. Currently, the red panda is listed as a single species consisting of two subspecies by the International Union for Conservation of Nature (IUCN) and is classed as Endangered with less than 10,000 remaining in the wild. In China, the red panda is listed as a Class II protected species with a reported population decline of 40% over the last 50 years [7]. Habitat destruction and fragmentation are the primary causes of the population decline; however, disease outbreaks, including parasitic infections, are an emerging threat for both the in situ and ex situ populations of the species. In particular, red pandas face parasitic threats from nematodes such as *Baylisascaris ailuri* and *Angiostrongylus vasorum* [8,9]. To our knowledge, no studies have reported natural infections of *Rhabditella* spp. in this host species, underscoring a significant gap in assessing its zoonotic potential and ecological role in parasite transmission.

This study aimed to identify nematodes isolated from captive Chinese red panda fecal samples using both morphological and molecular methods, elucidate their phylogenetic relationships through 18S-ITS-28S rDNA analysis, and evaluate the implications for captive wildlife management.

## 2. Methods

### 2.1. Sample Collection

Fresh fecal samples were taken from healthy Chinese red pandas housed at the Chengdu Research Base of Giant Panda Breeding (CRBGPB) located in Sichuan China under normal housing conditions for this institution [10]. All samples were collected from the enclosures, immediately labeled, and stored at 4 °C for subsequent analyses. Microscopic examination of fresh fecal samples was conducted within 24 h of collection. For parasite detection, the centrifugal sedimentation method was employed [11]. Briefly, fecal suspensions were prepared by homogenizing 1 g of feces in 10 mL of physiological saline (0.9% NaCl). The suspension was thoroughly mixed, followed by filtration through a 60-mesh sieve. The filtrate was centrifuged at 1500× *g* for 10 min. After discarding the supernatant, the pellet was resuspended in physiological saline and subjected to repeated centrifugation until the supernatant clarified. The final sediment was retained for microscopic analysis [1].

### 2.2. Nematode Isolation and Enrichment

Fecal samples positive for nematode adults and eggs via microscopy were selected for culture. Approximately 5 g of fecal material, adjusted to a semi-solid consistency, was placed on filter paper as a semicircular mound. To suppress fungal growth, 1 mL of 0.1% sodium carbonate (Na_2_CO_3_) was added to the dish. Cultures were incubated in a humidified, light-protected environment at 26 °C for 10 days [1,12]. Nematode larvae were subsequently enriched using the Baermann funnel technique [11] and examined microscopically.

### 2.3. DNA Amplification and Sequencing

Genomic DNA was extracted from cultured and enriched samples using the QIAamp^®^ Fast DNA Stool Mini Kit (QIAGEN N.V., Venlo, Germany), following the manufacturer’s protocol. Five overlapping PCR amplicons spanning the 18S-ITS-28S rDNA locus were generated using primers synthesized by Sangon Biotech (Shanghai, China) [2]. Primer pairs [13,14,15,16,17,18] and thermal conditions are listed in Table 1. Each 25 µL PCR reaction contained 1 µL each of forward and reverse primers (10 µM), 2 µL DNA template, 12.5 µL PCR master mix (Takara Bio, Shiga, Japan), and 8.5 µL nuclease-free water. Thermal cycling conditions included initial denaturation at 95 °C for 3 min, followed by 45 cycles of denaturation at 94 °C for 60 s, annealing at primer-specific temperature (Ta) for 60 s, and extension at 72 °C for 90 s, followed by a final extension at 72 °C for 5 min. PCR amplicons were electrophoresed on 1.5% agarose gel at 120 V for 30 min, and visualized under UV light using a gel documentation system, and sequenced by Sangon Biotech (Shanghai, China). Consensus sequences were assembled using Geneious Prime^®^ (2023.2.1) and aligned against the GenBank database via NCBI BLAST (https://www.ncbi.nlm.nih.gov/BLAST, 8 April 2025).

### 2.4. Phylogenetic Analysis

Maximum likelihood (ML) phylogenies were reconstructed using Molecular Evolutionary Genetics Analysis version 12 (MEGA12.0) with the best-fit substitution model selected under the Bayesian Information Criterion (BIC). Bootstrap support values were derived from 1000 replicates for comparative analysis with sequences from GenBank.

## 3. Results

### 3.1. Morphological Features

**Figure 1:**
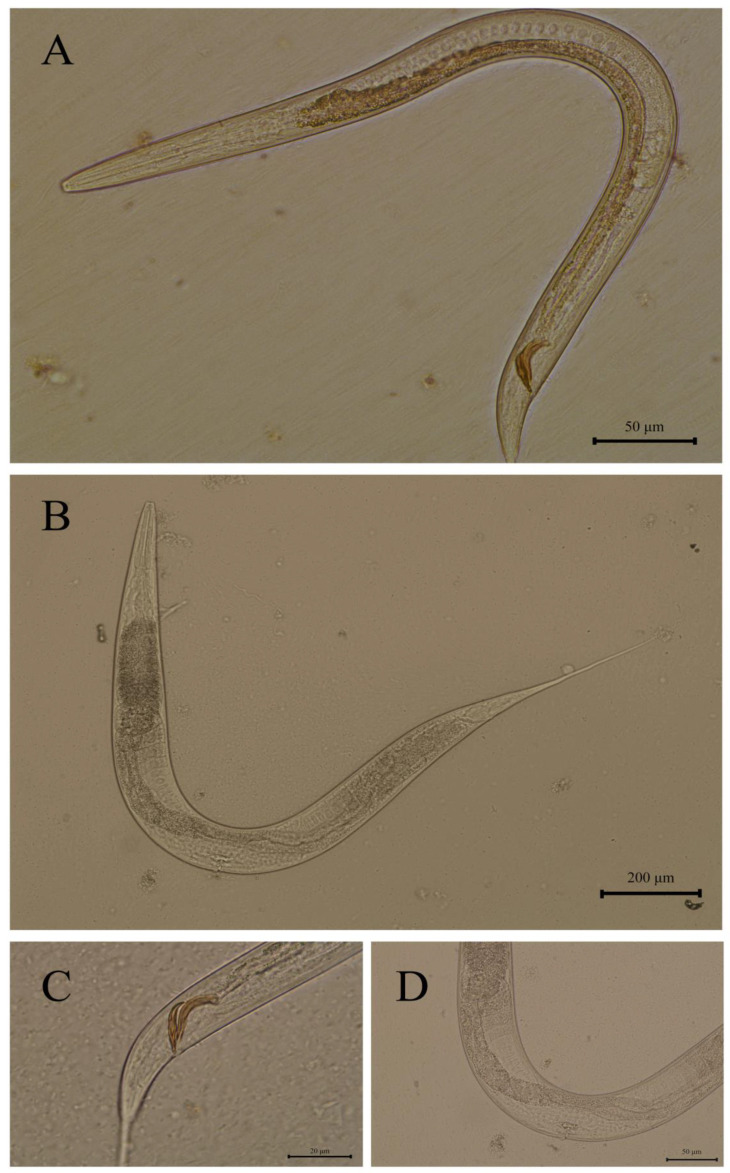
*Rhabditis axei* isolated from red panda feces in this study: (**A**) male, entire body; (**B**) female, entire body; (**C**) spicules and gubernaculum; (**D**) vulva.

Nematodes exhibited small body size and dioecious reproduction (Figure 1). The pharynx featured a cylindrical rhabditiform esophagus with distinct anterior and posterior esophageal bulbs. Male esophagus length ranged from 111 to 165 µm, while female esophagus length ranged from 162 to 218 µm (Figure 1A,B), and the buccal cavity depth ranged from 18.00 to 21.72 μm. Males displayed a basally swollen tail terminating in an elongated slender caudal end, with paired equal-length spicules (25–33 µm), that appeared spoon-shaped in lateral view with proximal curvature (Figure 1C). Females possessed an elongated, non-swollen tail with a midbody vulva (Figure 1D).

### 3.2. Molecular Detection

**Figure 2:**
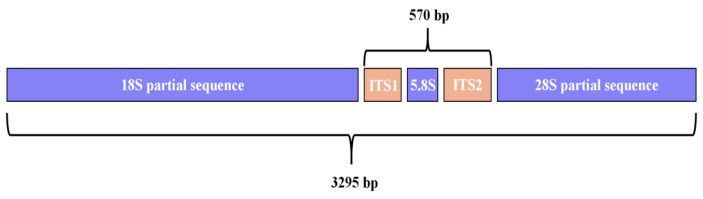
Map of contiguous sequence.

A contiguous 3295 bp DNA sequence (18S-ITS-28S) was assembled from PCR products, including partial 18S ribosomal RNA, internal transcribed spacer 1, 5.8S ribosomal RNA gene, internal transcribed spacer 2 (complete sequence), and partial 28S ribosomal RNA gene (Figure 2). BLAST analysis revealed 99.40% similarity with *R. axei* strains PP135624.1 and PP135622.1, and 99.34% similarity with PP135623.1. The contiguous DNA sequence determined in this study has been deposited in the GenBank database under accession number PV463385.

### 3.3. Phylogenetic Analysis

**Figure 3:**
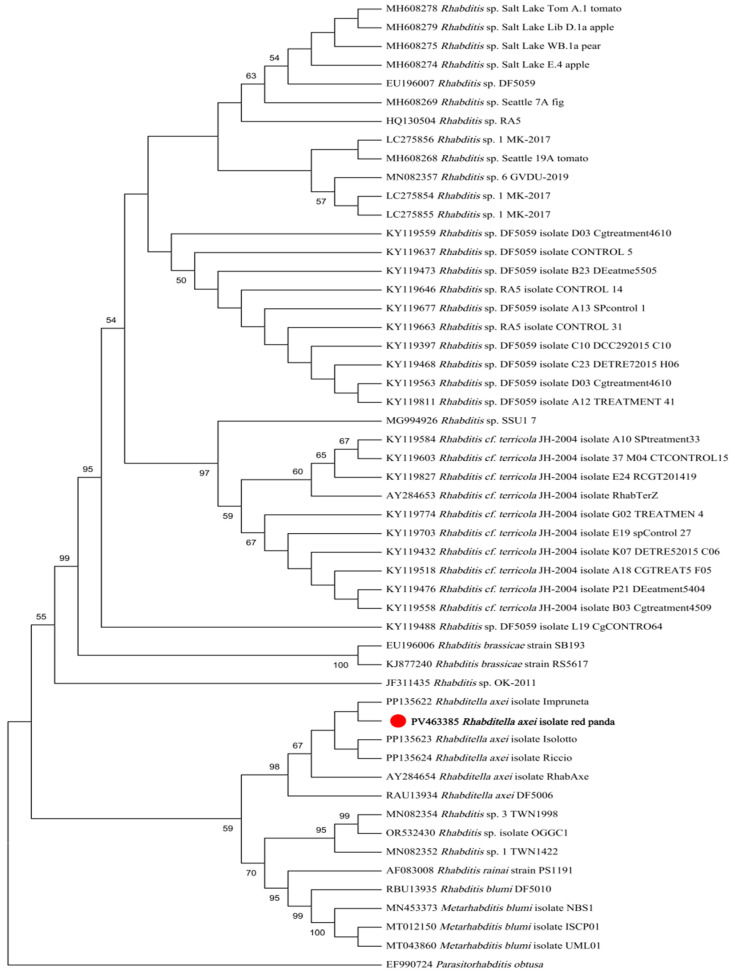
ML tree based on 18S gene fragments. The phylogeny was inferred using the maximum likelihood method and Tamura model of nucleotide substitutions, with bootstrap estimates from 1000 replicates. *Parasitorhabditis obtusa* was used as the outgroup. The percentage of replicate trees in which the associated taxa clustered together (1000 replicates) is shown next to the branches.

**Figure 4:**
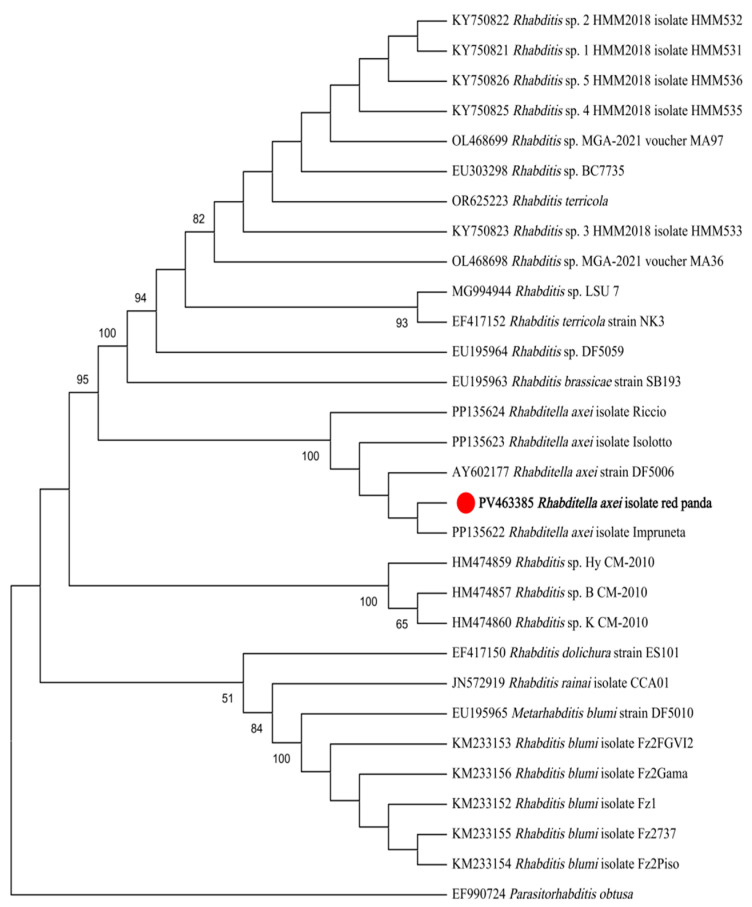
ML tree based on ITS gene fragments. The phylogeny was inferred using the maximum likelihood method and Kimura 2-parameter model of nucleotide substitutions, with bootstrap estimates from 1000 replicates. *Parasitorhabditis obtusa* as the outgroup. The percentage of replicate trees in which the associated taxa clustered together (1000 replicates) is shown next to the branches.

Maximum likelihood (ML) analysis based on 18S rDNA sequences demonstrated that the isolate formed a monophyletic clade with *R. axei* strains retrieved from GenBank, while remaining distinct from *R. blumi*, *R. brassicae*, *R.* cf. *terricola*, and unclassified *Rhabditella* spp. (Figure 3). Similarly, ML analysis based on ITS sequences revealed congruent topological relationships, with the isolate exclusively clustering with *R. axei* reference sequences and showing clear divergence from *R. dolichura*, *R. rainai*, *R. blumi*, *R. brassicae*, *R. terricola*, and other congeners (Figure 4).

## 4. Discussion

Rhabdiasidae (Railliet, 1915) nematodes typically exist as free-living organisms in decaying vegetation and organic-rich soil, exhibiting a wide distribution in nature. Their rhabditiform larvae feed on decomposing organic matter and develop into adults, demonstrating high adaptability within specific ecological niches. Some species occasionally parasitize animals, including humans [1]. The detection of *R. axei* in Chinese red pandas, a species with no prior records of rhabditid infection, suggests broader environmental adaptability than previously recognized. This finding aligns with studies highlighting the opportunistic nature of *Rhabditella* spp., which exploit ecological niches where host defenses are suboptimal.

As a free-living nematode primarily inhabiting sewage and decaying vegetation, *R. axei* may incidentally infect humans or animals through contaminated water or direct contact. Levine et al. (1963) reported heavy infestations in canine feces and perianal fur [19], while Azazy et al. (1988) observed the nematode in caged poultry feces [20]. Uotani (1960) demonstrated through rodent experiments that *R. axei* can penetrate intestinal tissues and migrate to internal organs [21]. Human infections resulting from ingestion or urogenital contact with contaminated water have been documented [3,4,5,21]. Sciandra et al. reported [2] *R. axei* in insects (*Paysandisia archon*) and mollusks (*Cornu aspersum*), suggesting it may proliferate in carcasses or act as a pseudoparasite. While other Rhabditis nematodes infect insects and mollusks via symbiotic bacteria that induce lethal sepsis—decomposing the host cadaver to support nematode reproduction—R. axei employs direct tissue invasion in mammals without bacterial co-pathogenesis [22].

The facultative parasitic capabilities of free-living nematodes, as exemplified by *R. axei* in this study, reflect adaptive evolutionary strategies to dynamic environments. Similar behaviors are documented in *Strongyloides stercoralis* and *Pelodera strongyloides*, which transition from saprophytic to parasitic life cycles under conducive conditions [23]. The ability of *R.* spp. to exploit both saprophytic and parasitic niches underscores their ecological versatility and highlights the need for heightened surveillance for the conservation of wildlife, both in situ and ex situ as well as the potential to mitigate zoonotic risks.

Repeated parasitic infections in captive wild animals may lead to illness or death [12]. Parasites are an important disease affecting both the wild and captive red panda populations [8]. Currently, more than 20 species of parasites have been identified in red pandas, among which nematodes pose significant threats [9,11]. *Canine dirofilariosis* has caused fatalities in more than 10 captive red pandas [24]; nematodes such as *Baylisascaris ailur*, *Aelurostrongylus sinica*, and *Angiostrongylus vasorum* have also contributed to morbidity and mortality in these species [8]. Parasitic infections pose a substantial threat to the sustainability of the captive red panda population and its conservation in general; therefore, advancing research on red panda parasitosis is crucial for the management of this endangered species.

This study reports the first detection of *R. axei* in red panda feces, expanding the known host range of this facultative parasitic nematode. These findings underscore the utility of integrative morphological–molecular approaches in nematode taxonomy and highlight the potential for environmental exposure to drive accidental parasitism in non-traditional hosts. Notably, adult *R. axei* may be confused with *Enterobius vermicularis* [23] or *Strongyloides stercoralis* [3], while its eggs resemble those of hookworm [25]. Previous *Strongyloides* sp. infections in red pandas warrant particular attention due to their similar free-living parasitic life cycles. Therefore, molecular confirmation should complement morphological identification for accurate diagnosis.

The morphological features observed in the isolated nematodes included anterior and posterior esophageal bulbs. Both sexes possessed elongated tails, with males exhibiting a basally swollen tail that terminated in an elongated slender caudal end and bore paired equal-length spicules with spatulate morphology in lateral view. Females displayed a non-swollen elongated tail with a midbody vulva. These morphological characteristics suggest that the nematodes isolated in this study may belong to the genus *Rhabditella* [1]. The male’s spoon-shaped spicules with proximal curvature and the female’s midbody vulva position closely match prior descriptions of this species. These traits, combined with the absence of morphological deviations from established *R. axei* profiles, strongly support its identification. Notably, the consistency between morphological and molecular data minimizes ambiguity in species delineation, a critical consideration given the high phenotypic plasticity within *R.* spp. [3,23,25].

Molecular analyses further corroborated the identification, with assembled 18S-ITS-28S rDNA sequences exhibiting >99% similarity to *R. axei* reference strains. Phylogenetic analyses based on 18S and ITS rDNA gene sequences both robustly clustered the isolate within *R. axei* specific clades. The concatenated 18S-ITS-28S locus proved highly effective in resolving the phylogenetic position of the isolate.

The small subunit ribosomal RNA (SSU rRNA) of eukaryotes, encoded by chromosomal genes and referred to as 18S rDNA, exhibits minimal evolutionary divergence in its primary and secondary structural organization. As one of the most conserved DNA sequences discovered to date, 18S rDNA retains a high abundance of conserved and semi-conserved nucleotide regions, making it an invaluable molecular marker for investigating phylogenetic relationships among higher taxonomic groups. It has been widely utilized in the molecular systematics of metazoans. Fitch et al. (1995) [26] employed 18S rDNA sequences to explore the phylogenetic relationships of six nematode species across nine genera within the family *Rhabditidae*. Similarly, Xue et al. (2024) [27] analyzed 18S rDNA sequences from soil nematodes to elucidate their evolutionary affiliations.

In eukaryotes, the internal transcribed spacer (ITS) regions, situated between 18S rDNA and 28S rDNA, are non-coding sequences characterized by low selective pressure and high sequence variability. As moderately conserved regions, ITS serves as a critical molecular marker for taxonomic and phylogenetic studies at species and subspecies levels. For instance, ITS-based analyses have been extensively employed in the classification and phylogenetic reconstruction of various nematodes [28,29]. This dual-marker approach leverages the complementary strengths of conserved and hypervariable regions, enhancing resolution in clades with limited morphological divergence. The monophyletic clustering of the isolate with *R. axei* references across both gene trees underscores the reliability of this locus for species-level identification in rhabditid nematodes.

The infection route in Chinese red pandas remains speculative but may involve environmental contamination or predation on intermediate hosts. The habitat of captive red pandas at the CRBGPB includes outdoor naturally forested enclosures containing water reservoirs, local vegetation, and soil, with wild access to birds, insects, and mollusks [10]. Red pandas primarily consume bamboo; however, they will occasionally eat small invertebrates; ingestion of nematode-laden soil or arthropods could introduce *R. axei* into the digestive tract. Alternatively, contact with contaminated water or foliage in captive enclosures might promote cutaneous or oral transmission. Further studies mapping nematode distribution in captive and wild habitats, alongside host behavioral analyses, are needed to clarify transmission dynamics.

While wildlife typically exhibit high parasite prevalence with low morbidity, immunocompromised individuals become vulnerable to pathogenic effects. Nematode migration may cause mechanical damage to blood or lymphatic vessels [12]. Human infections often present subclinical gastrointestinal manifestations, though urinary system involvement may lead to hematuria and proteinuria [3,4,5,21]. The asymptomatic presentation in red pandas may reflect low parasite burden or localized infection, but potential health impacts necessitate monitoring. The detection of *R. axei* in Chinese red panda feces may represent an incidental infection, given the absence of overt clinical symptoms in the affected individuals. This observation raises questions regarding the transient nature of the infection and the host–parasite interaction dynamics. While *R. axei* is known to exhibit facultative parasitism, its transmission pathways in red pandas—whether through environmental contamination, intermediate hosts, or direct contact—remain speculative. Furthermore, the pathogenicity of *R. axei* in this novel host is unclear, as subclinical infections may reflect either low virulence or effective host immune responses. Comparative studies in other mammals suggest that clinical outcomes depend on infection intensity and tissue tropism; however, such parameters remain unquantified in red pandas. Future investigations should prioritize longitudinal surveillance to assess infection prevalence, experimental models to elucidate transmission routes, and histopathological analyses to evaluate tissue-specific damage. These efforts are critical to understanding the ecological and health implications of *R. axei* in endangered wildlife populations such as the red panda and mitigating potential zoonotic spillover risks.

## 5. Conclusions

This study provides the first evidence of *R. axei* in in Chinese red pandas, validated through morphological, molecular, and phylogenetic analyses. The findings emphasize the zoonotic potential of free-living nematodes and underscore the importance of environmental hygiene in captive animal management. Subclinical infections in Chinese red pandas suggest either low virulence or effective host immunity, necessitating longitudinal studies to evaluate pathogenicity and transmission routes. Future research should prioritize epidemiological surveys in captive and wild populations of red pandas, experimental models to elucidate tissue tropism, and genomic comparisons to assess zoonotic spillover risks. These efforts are critical for red panda conservation and mitigating emerging parasitic threats at human–animal interfaces.

## Figures and Tables

**Table 1:** Primers used for PCR and sequencing.

Primers	Direction	Sequence (5′-3′)	Tm (°C)	rRNA Gene	Reference
988F	F	CTCAAAGATTAAGCCATGC	45	18S	[13]
1912R	R	TTTACGGTCAGAACTAGGG	45	18S	[13]
1813F	F	CTGCGTGAGAGGTGAAAT	45	18S	[13]
2646R	R	GCTACCTTGTTACGACTTTT	45	18S	[13]
nem1	F	GCAAGTCTGGTGCCAGCAGC	55	18S, ITS	[14]
nem2	R	CCGTGTTGAGTCAAATTAAG	55	18S, ITS	[14]
18S-F	F	TTGATTACGTCCCTGCCCTTT	55	ITS	[15]
28Srev430	R	CAACTTTCCCTCACGGTACTTGT	55	ITS	[16]
D2F	F	CCTTAGTAACGGCGAGTGAAA	55	ITS, 28S	[17]
D3B	R	TCGGAAGGAACCAGCTACTA	55	ITS, 28S	[18]

## Data Availability

The data used in this article are available on request by contacting the corresponding authors.
